# Comparative Proteomics of Potato Cultivars with a Variable Dormancy Period

**DOI:** 10.3390/molecules27196621

**Published:** 2022-10-05

**Authors:** Daniel Mouzo, Raquel Rodríguez-Vázquez, Carlos Barrio, Lucio García, Carlos Zapata

**Affiliations:** 1 Department of Zoology, Genetics and Physical Anthropology, University of Santiago de Compostela, 15782 Santiago de Compostela, Spain; 2 Meat Technology Center of Galicia, 32900 San Cibrao das Viñas, Spain

**Keywords:** dormancy, endodormancy, paradormancy, *Solanum tuberosum*, oxylipins, α-ketol, catalase, hydrogen peroxide, lipid mobilization

## Abstract

The control of the duration of the dormancy phase is a significant challenge in the potato industry and for seed producers. However, the proteome landscape involved in the regulation of the length of the dormancy period over potato cultivars remains largely unexplored. In this study, we performed for the first time a comparative proteome profiling of potato cultivars with differential duration of tuber dormancy. More specifically, the proteome profiling of Agata, Kennebec and Agria commercial potato varieties with short, medium and medium-long dormancy, respectively, was assessed at the endodormancy stage using high-resolution two-dimensional electrophoresis (2-DE) coupled to reversed-phase liquid chromatography–tandem mass spectrometry (LC-TripleTOF MS/MS). A total of 11 proteins/isoforms with statistically significant differential abundance among cultivars were detected on 2-DE gels and confidently identified by LC-TripleTOF MS/MS. Identified proteins have known functions related to tuber development, sprouting and the oxylipins biosynthesis pathway. Fructokinase, a mitochondrial ADP/ATP carrier, catalase isozyme 2 and heat shock 70 kDa were the proteins with the strongest response to dormancy variations. To the best of our knowledge, this study reports the first candidate proteins underlying variable dormancy length in potato cultivars.

## 1. Introduction

**Figure 1:**
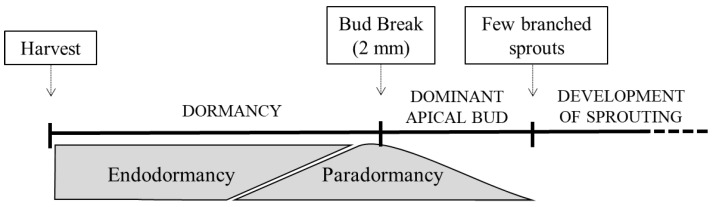
Schematic timeline from harvest to tuber sprouting under favorable conditions. Endodormancy is the first stage of tuber dormancy where the growth of meristems is inhibited. Once endodormancy weakens, there is a transition to paradormancy, and the apical bud will grow while the other meristems will be inhibited in paradormancy. Subsequently, the release of the paradormancy, allows for the appearance of secondary shoots that together with the apical bud form the plant through normal sprouting. Furthermore, endodormancy will become ecodormancy, and growth will be inhibited under unfavorable environmental conditions (e.g., low temperatures).

The potato (*Solanum tuberosum* L.) is the fourth most widely grown crop in the world after maize *(Zea mays* L.), wheat (*Triticum aestivum* L.) and rice (*Oryza sativa* L.). However, the efficiency of production of the potato (2011 kg/ha) exceeds that of the first three crops as a whole [1]. The post-harvest storage of potatoes is a crucial part of the potato production cycle to satisfy consumer and processing industry demands throughout the year [2,3,4]. The preservation of tuber quality in a fully hydrated form during extended storage requires preventing tuber sprouting following postharvest physiological dormancy [5]. Endodormancy, paradormancy and ecodormancy are the three dormancy stages occurring during tuber development after tuber harvest (Figure 1). Paradormancy and ecodormancy take place after the endodormancy stage when external factors are favorable and unfavorable, respectively [5,6,7,8]. Dormancy release is initiated by a 2 mm long apical bud meristem sprouting with apical dominance [9,10,11,12].

Physical and chemical agents are currently used for controlling the length of the dormancy period. One of the most usual practices is to keep the harvested potatoes at a low storage temperature, delaying the germination of the apical bud [13,14]. Phytotoxic chemical sprout suppressors, such as chlorpropham (CIPC), dimethylnaphthalene and maleic hydrazide, are also used to delay the exit of dormancy [6,15]. These practices, however, are not innocuous and can affect the quality of the tuber. Thus, low storage temperature can lead to cold-induced sweetening that causes the conversion of starch into reducing sugars [16]. The accumulation of reducing sugars leads to the brown-pigmented color of potato chips and potentially increases the amount of cancer-causing acrylamide as a by-product of the Maillard reaction [17,18,19]. Furthermore, the CIPC is a phytotoxic chemical widely used in the potato industry that inhibits mitosis and modifies spindle formation in the meristems, causing damage to the eyes of the tuber [20,21]. In addition, the new legislation and the pressure from retailers for a reduction of chemical compounds in food production requires the development of alternative technologies to control the duration of the dormancy phase [21].

The length of the dormancy period of potato tubers seems to be extremely variable depending on the genotype as well as the growth and storage conditions, varying between 15 and 197 days [13,14,22,23,24,25,26,27]. A number of studies have shown, however, that the potato genotype is the main factor that affects the duration of dormancy length [6,12,14,28,29]. Simmonds [30] showed that the duration of dormancy is strongly influenced by a polygenetic component. Subsequently, studies by Freyre et al. [31] and van den Berg et al. [32] in populations of potatoes and potato hybrids concluded through quantitative trait loci (QTL) mapping that there was a direct genetic control of potato tuber dormancy. However, these QTL analyses failed to reveal which specific genes were involved [5].

Proteomics provides a wide spectrum of powerful molecular tools for the high throughput systematic analysis of the dynamic changes of protein expression enabling the elucidation of the regulatory networks modulating cellular processes such as the control of the length of the dormancy period. A number of targeted and non-targeted proteomic studies have contributed to deciphering temporal changes in the proteome and phosphoproteome throughout the tuber life cycle stages by using gel-based and gel-free technologies [9,28,33,34,35]. These temporal proteomic studies enabled the identification of proteins and phosphoproteins linked to the different phases of tuberization and a deeper understanding of the crosstalk of proteins involved in tuber life stage transitions, including the endodormancy-to-sprouting transition. However, the proteomic studies carried out to date in potato tubers cannot provide specific information on the subproteome involved in the control of dormancy length variations because they were designed for a completely different purpose.

In this study, a comparative proteome profiling of three commercial potato cultivars (Agata, Kennebec and Agria) with differential durations of tuber dormancy (short, medium and medium-long, respectively) was conducted at the endodormancy stage from tubers of each cultivar grown under similar conditions in the same experimental field. Proteome profiles were assessed by 2-DE coupled to LC-TripleTOF MS/MS. This is the first study with an experimental approach addressed in particular to unraveling the sub-proteome and candidate proteins underlying dormancy length variations in potato cultivars.

## 2. Results and Discussion

### 2.1. Dormancy length Variation between the Cultivars Studied

Table 1 shows the length of dormancy period of Agata, Kennebec and Agria potato tubers used in this study. The results reveal that qualitative differences in dormancy length between cultivars grown in the same experimental field are in agreement with previous reports on those same three cultivars grown under different environmental conditions [36,37]. Thus, mean (±SE, standard error) values of the length of the dormancy period in Agata, Kennebec and Agria cultivars were 15.3 ± 0.3 (short dormancy), 53.5 ± 2.0 (medium dormancy) and 62.0 ± 3.0 days (medium-long dormancy), respectively (Table 1). Our observations therefore add to the existing experimental evidence suggesting that although sprouting is a complex process where environmental and genetic factors interact in a coordinated manner, the genotype is the major factor that affects the duration of dormancy [28,29,35]. Most importantly for the purpose of our study, the potatoes from the three cultivars were grown under homogeneous environmental conditions and meet the requirements to address the proteomic changes involved in dormancy length variations. This comparative proteomic study was performed immediately following harvest, facilitating and unraveling the sub-proteome linked to dormancy length variations among potato cultivars. Note that the evolution of the dormancy in the studied cultivars is very different. Therefore, the comparison in other tuber life cycle stages might make it difficult to differentiate changes in the proteome directly or indirectly attributable to differences in dormancy among cultivars from background changes of the proteome unrelated to dormancy.

### 2.2. Differential Tuber Proteome Profiles between Cultivars

**Figure 2:**
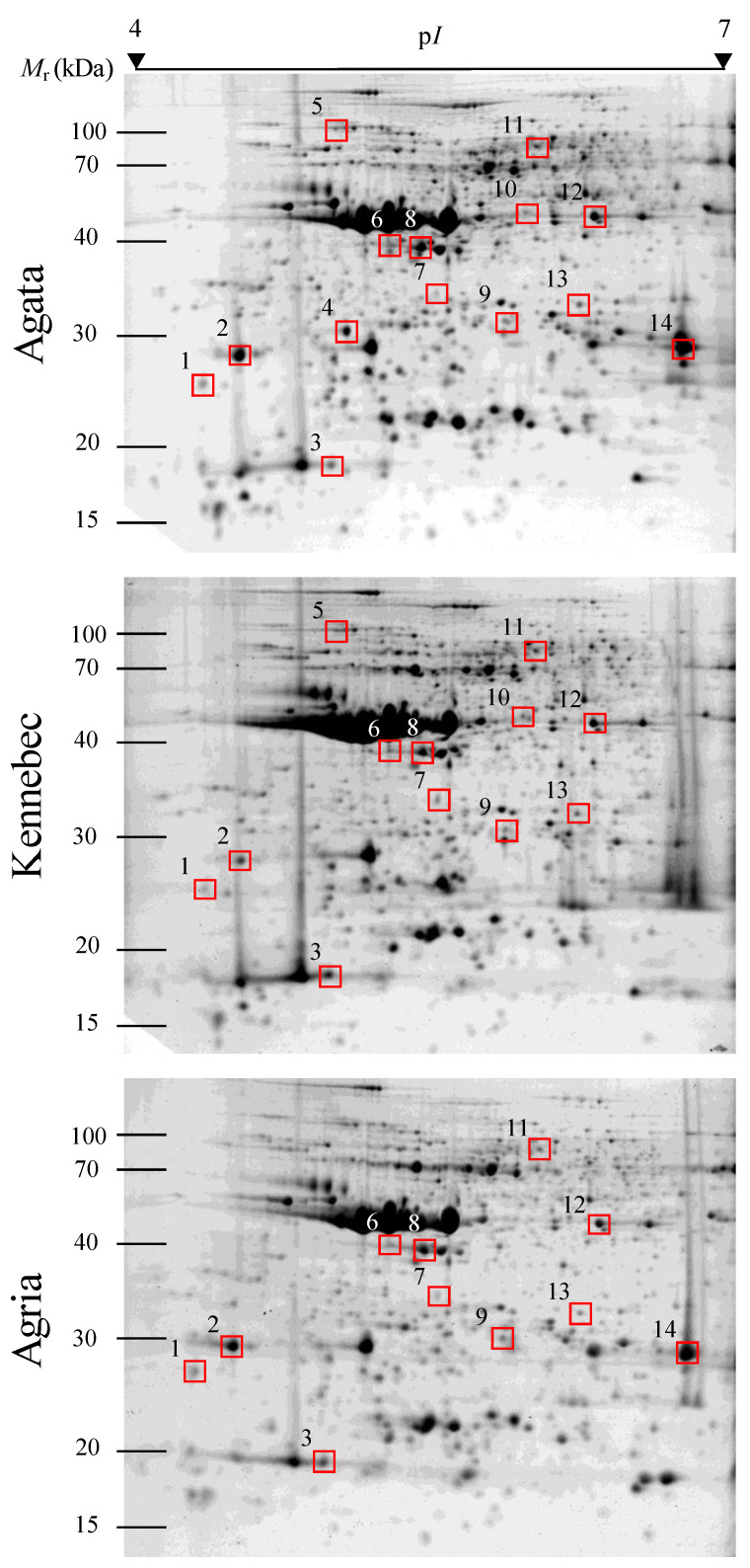
Two-dimensional electrophoresis gel images of global endodormancy proteomes from three potato cultivars (Agata, Kennebec and Agria) at the endodormancy stage. Squares indicate the fourteen spots that house a single differentially abundant protein between cultivars according to LC-TripleTOF MS/MS analysis.

Representative proteomic 2-DE profiles from tubers of Agata, Kennebec and Agria potato cultivars at the endodormancy stage are shown in Figure 2. Furthermore, 2-DE profiles of potato tubers across replicates (1–4) of each cultivar are shown in Supplementary Figure S1. Gel images indicate that high-resolution profiles of tuber proteins were obtained, with well-defined and highly reproducible spot patterns, allowing for reliable detection, matching and quantification of spot volumes over cultivars by the PDQuest software. A saturated gel zone (p*I* = 4.8–5.3 and *M*r = 45–51 kDa) is due to the protein patatin, the major potato storage protein, that accounts for up to 45% of the total soluble protein [35].

**Figure 3:**
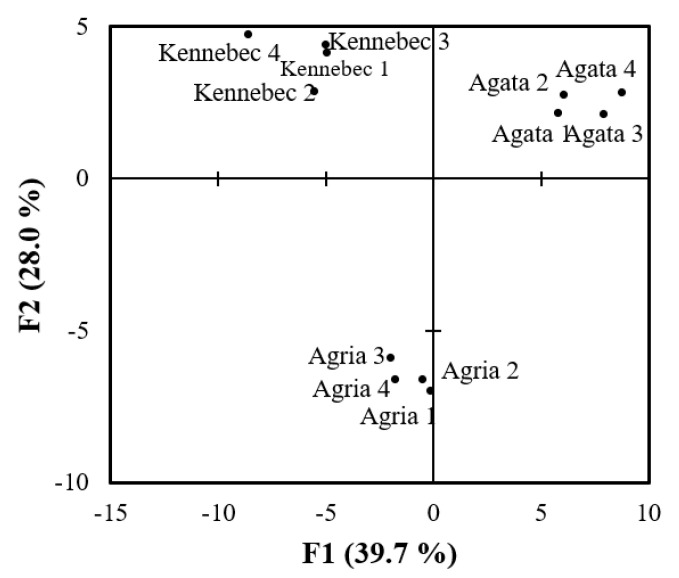
Principal component analysis (PCA) using the volume of 78 differentially abundant (*p* < 0.05) spots between Agata, Kennebec and Agria (replicates 1–4) potato cultivars at the endodormancy stage.

A total of 345 reproducible protein spots present in at least three of the four replicates of each cultivar were identified through gel image analysis with PDQuest software. Finally, 78 out of 345 (22.6%) spots showed statistically significant volume differences between pairs of cultivars (*p* value < 0.05) using 95% bootstrap CIs calculated by the bias-corrected percentile method and the Bonferroni correction (data not shown). Principal component analysis (PCA) with differentially abundant protein spots disclosed that the first two principal components (68% of the total variation) enable separate clustering of all the replicates of each cultivar (Figure 3). Taken together, the results indicate that 2-DE is able to detect remarkable proteomic differences among the three cultivars with differential dormancy length.

### 2.3. Identification of Dormancy Length-Dependent Candidate Proteins

Protein spots with statistically significant differential abundance between cultivars were selected for LC-TripleTOF MS/MS analysis. In total, 52 of the 78 spots (67%) were confidently identified (Supplementary Table S1). However, most spots (38) contained mixtures of two or more proteins. One of the main criticisms against the use of 2-DE for quantitative proteomics is the comigration of proteins on the gels, resulting in spots that may contain more than one protein. Consequently, these spots were excluded for subsequent analysis addressing quantitative protein differences among cultivars. Protein identifications for the remaining 14 spots housing a single protein are listed in Table 2. The challenge is to assess whether proteins with changes in abundance among cultivars identified by MS have some functional relationship(s) with dormancy length variations and are not only proteomic changes of background without relation to dormancy. This key step in proteomic workflows was tackled through an intensive search using different bioinformatic tools [39], protein databases (e.g., UniProt/SwissProt database) and an in-depth literature review.

Four protein spots corresponded to different isoforms of mitochondrial ADP/ATP carrier proteins (AAC) involved in the exchange between matrix ATP generated during oxidative phosphorylation and the cytosolic ADP [40,41,42]. Note that AAC isoforms exhibited remarkable variations in p*I* (from 4.6 to 6.4) and minor changes in *M*r (from 30.5 to 32.4) on 2-DE gels (Figure 2 and Table 2). The *pI* and/or *M*r of proteins could be altered by post-translational modifications (PTMs), such as phosphorylations, with the addition of negatively charged phosphate groups replacing hydroxyl groups in amino acid residues [43]. Experiments in progress in our laboratories are trying to elucidate whether they are differentially phosphorylated isoforms by their content in ADP and ATP, using multiplex identification of phosphoproteins with Pro-Q Diamond Phosphoprotein stain coupled to 2-DE [43]. Protease inhibitors were also highly represented. More specifically, two serine and one aspartic protease inhibitors were identified. Both proteins possess defense activities against pathogens, and their differential abundance could be influenced by the presence of pathogens. Fernández et al. [44] found an overexpression of these protease inhibitors after infecting tubers with *Phytophthora infestans*. In addition, Bartová et al. [45] described the reduction of several potato pathogens through treatment with these proteins. Due to the potential external influence, these protease inhibitors were excluded from further analyses. Catalase 2 was another protein identified in this study, which belongs to the catalase family (CAT). This enzyme reduces the 65% of hydrogen peroxide (H_2_O_2_) that is generated, along with other reactive oxygen species (ROS), during tuber dormancy, to 2H_2_O + O_2_. Meanwhile, the rest of H_2_O_2_ is reduced by ascorbate peroxidase and glutathione peroxidase ROS-scavenging enzymes [46,47]. Another protein identified was the enzyme linolate 9S-lipoxygenase 2 belonging to the lypoxygenases (LOX) family, which is responsible for the synthesis of oxylipins through the oxidation of polyunsaturated fatty acids (PUFAS) [48]. This enzyme synthesizes the (9S,10E,12Z)-9-hydroperoxy-10,12-octadecadienoic acid (9-HPODE), the predominant LOX product in the potato tuber [49]. The list of proteins identified is completed with the translationally controlled tumor protein homolog (TCTP), frucktokinase (FRK), heat shock protein 70 kDa, patatins and actin-82. These proteins have various activities such as microtubule organization, catalysis of the fructose phosphorylation, mitochondrial chaperones, storage functions and structural proteins, respectively [9,45,50,51,52,53,54].

**Table 2:** List of spots hosting a single protein with statistically significant differential abundance (*p* < 0.05) between pairs of potato cultivars that were identified by LC-TripleTOF MS/MS.

Spot Code	Unused ProtScore	% Protein Confidence	Sequence Cov. (%)	Peptides (95%)	Accession	Protein Name	p*I* (th/ obs)	*M*_r_ (kDa) (th/obs)
1	3.63	>99	45.24	0	P43349	Translationally controlled tumor protein homolog	4.6/4.1	18.8/26.1
2	5.28	>99	22.04	3	P58515	Serine protease inhibitor 2	4.9/4.2	20.1/28.4
3	2.53	>99	30.11	2	P58515	Serine protease inhibitor 2	4.9/4.5	20.1/18.0
4	1.92	99	11.92	3	P25083	ADP, ATP carrier protein, mitochondrial	9.8/4.6	42.1/30.5
5	1.37	96	1.17	1	Q08276	Heat shock 70 kDa protein, mitochondrial	5.4/4.6	73.1/98.4
6	1.53	97	8.02	0	Q3YJT3	Patatin-2-Kuras 1	5.1/5.0	41.1/39.8
7	3.86	>99	14.25	1	P25083	ADP, ATP carrier protein, mitochondrial	9.8/5.3	42.1/33.6
8	4.51	>99	14.42	2	P37829	Fructokinase	5.5/5.2	33.8/39.3
9	6.00	>99	9.07	3	P25083	ADP, ATP carrier protein, mitochondrial	9.8/5.8	42.1/31.2
10	2.00	99	3.10	1	Q3YJT5	Patatin-05	5.4/6.0	42.5/42.0
11	18.26	>99	19.16	9	O24379	Linoleate 9S-lipoxygenase 2	5.4/6.1	97.1/85.3
12	4.99	>99	12.20	2	P55312	Catalase isozyme 2	6.6/6.5	56.5/41.6
13	2.10	99	17.62	1	P25083	ADP, ATP carrier protein, mitochondrial	9.8/6.4	42.1/32.4
14	3.74	>99	44.09	4	P17979	Aspartic protease inhibitor 8	6.5/7.0	24.2/28.9

Unused ProtScore is a measure that reflects the unique peptides to a given protein. The protein confidence threshold was set higher than 95% by the following formula Unused ProtScore = −log (1–% confidence/100). Sequence coverage (%) is the proportion of amino acids that match the peptides identified, with a confidence level higher than 0 and subsequently divided by the total amino acids in the sequence. Peptides (95%) indicate the number of peptides identified with at least 95% confidence [55,56].

### 2.4. Quantitative Changes of Candidate Proteins between Cultivars

A total of 11 proteins/isoforms were eventually selected for the quantitative analysis of protein changes between pairwise cultivars after excluding defense proteins against pathogens. The mean (±SE) of spot volumes over replicates for each selected protein and cultivar, as well as statistically significant differences between pairs of cultivars, assessed by 95% bootstrap CIs corrected with the Bonferroni method, are shown in Supplementary Table S2. The quantitation of statistically significant changes of protein abundance (*p* value < 0.05) between potato cultivars was assessed by *FC* and *RC* statistics (Table 3). It can be seen that *FC* is less useful than *RC* as a general measure of quantitative changes of proteins among sample groups, as previously shown [39,42,57,58]. The *FC* measure takes values of −∞ or +∞ for unshared (or unique) protein spots among sample groups, whatever their difference in volume. Note that *RC* is a more intuitive measure than *FC* because it always ranges between −1.0 and +1.0 and is capable of quantifying differences in protein abundance between shared and non-shared proteins.

Table 3 shows that the extent of quantitative changes in protein abundance underwent remarkable variations between pairs of cultivars. Thus, absolute *RC* values ranged from 0.068 (AAC, cvs Agata–Kennebec) to 1.0 (FRK, cvs Agata–Kennebec). FRK was the protein with the most pronounced quantitative change between cultivars (*RC* = −1.0, cvs Agata–Kennebec), followed by AAC (*RC* = −0.489, cvs Agata–Kennebec and Agata–Agria), CAT-2 (*RC* = −0.391, cvs Agata–Kennebec) and mt-HSP70 (*RC* = −0.311; cvs Kennebec-Agria). Therefore, they were the candidate proteins with the strongest response to dormancy length variations over cultivars.

The sign (negative or positive) of *RC* indicates the cultivar where candidate proteins were more abundant in pairwise comparisons. TCTP and FRK proteins were more abundantly represented in the Agria (short-dormancy) cultivar than in the Kennebec (medium-dormancy) cultivar (*RC* values < 0). TCTP is a plant growth regulator component of the TOR (target of rapamycin) signaling pathway involved in the regulation of growth [59]. It was shown that the silencing of TCTP expression in *Arabidopsis thaliana* by RNA interference (RNAi) slows vegetative growth [60]. Furthermore, the regulation of FRK activity affects potato tuber metabolism and plays an important role in maintaining a balance between sucrose synthesis and degradation [54]. The amount of most isoforms of ACC protein (spots 4, 7 and 9) changed significantly only in pairwise comparisons with Agata, the cultivar with the shortest dormancy. It has been reported that a slight reduction of AAC expression by antisense RNA leads to a sharp decrease in tuber yield [41]. Finally, the enzyme 9-LOX was also overrepresented in cv Agria. It is the most predominant member of the LOX family of proteins in potato tubers [61]. Jasmonate precursors are one of the products derived from LOX that are involved in the regulation of tuber growth. According to this, deletion of the Lox1 gene results in tubers with yield reduction and an abnormal morphology [62].

We also performed a prospective gel-free study from total protein extracts of each potato cultivar (Agata, Kennebec and Agria) using the data-independent acquisition-based SWATH-MS [63] technology (data not shown). In total, 500 proteins showed differential abundance (*p* < 0.05) among cultivars. Of these 500 proteins, 255 were uncharacterized proteins. Nevertheless, only 23 of the 245 characterized proteins were confidently identified after using the Bonferroni correction for multiple significance tests, a number of proteins close to those identified with 2-DE. In addition, the list of proteins identified by SWATH-MS was only partially overlapping with that of the 2-DE-based proteomic study by including the proteins AAC, FRK, CAT and Patatin. This result is in agreement with comparative studies between 2-DE-based and gel-free MS-based proteomic approaches, suggesting that both types of techniques are complementary and are used together to provide a more complete proteome coverage [64,65,66]. By way of illustration, a comparative analysis between 2-DE and gel-free multidimensional protein identification technology (MudPIT) in rice showed that 29% of the proteins identified were unique to the 2-DE approach [64]. No relationship of the remaining non-overlapping proteins identified by SWATH (i.e., 16 of 23 proteins) with dormancy length variations in potato was found, using the above-mentioned search tools.

### 2.5. Hypothetical Molecular Mechanism of Dormancy Length

**Figure 4:**
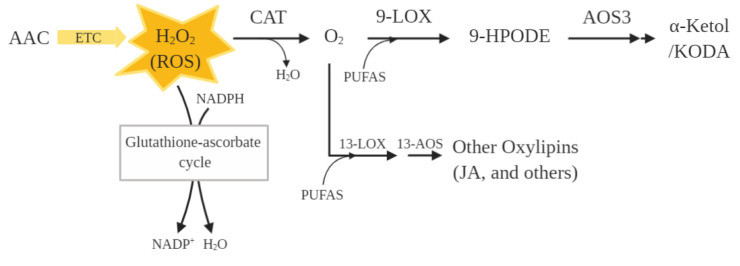
Simplified scheme of differentially abundant proteins between potato tubers from cultivars at the endodormancy stage involved in oxylipin biosynthesis.

Some of the proteins identified in this study participate in the biosynthesis pathway of the oxylipins (Figure 4). This result suggests that oxylipin biosynthesis might be involved in the regulation of dormancy length variations. First, AAC transports ADP into the mitochondrial matrix for ATP synthesis, which is the main source of ROS (e.g., superoxide (O_2_-) and H_2_O_2_) [67]. In addition, O_2_- is transformed into H_2_O_2_ by superoxide dismutase, which remains constant during dormancy [68]. H_2_O_2_ is one of the most important ROS in the tuber and can be harmful to the cell, although it is also known to have activities associated with the development of the plant [68,69]. Varieties with a higher amount of AAC lead to a high potential abundance of ROS, increasing the expression of CAT that can cope with oxidative damage. O_2_ is a ROS product of the CAT reaction with high migration capacity and the ability to form endoperoxides or hydroperoxides [69,70]. This O_2_ is subsequently used by the LOX family for the oxidation of PUFAS, synthesizing the oxylipin family [62,71]. The product of 9-LOX is oxylipin 9-HPODE, the highly specific substrate of the enzyme allene oxide synthase 3 (StAOS3). The main product of these chain reactions is α-ketol, also named KODA in other species [72,73,74,75]. It has been speculated that α-ketol could play a role in the regulation of tuber growth [62]. This assumption is tempting since oxygenated storage lipids not only increase during germination, but are also preferentially cleaved, initiating the mobilization of storage lipids [76]. Moreover, the expression of α-ketol is fivefold higher in the sprouting eyes of the potato than any other oxylipin [73]. Sakamoto et al. [75] also showed that α-ketol promotes the endodormancy release in buds of the Japanese pear flower (*Pyrus pyrifolia Nakai*). It is also known that α-ketol alters seed physiology. In this regard, treatments with α-ketol increase seed wheat germination even in drought conditions [74].

This proteomic study has been carried out on tubers at the endodormancy stage, where increased CAT activity is expected [77]. However, CAT isoenzymes are downregulated when the dormancy period ends and only the glutathione-ascorbate pathway is responsible for H_2_O_2_ reduction [46,77,78]. This causes an increase in H_2_O_2_, which has been correlated with dormancy release. It was possible to remove the tuber from dormancy using 1% thiourea (CAT inhibitor) and/or by supplying H_2_O_2_ [68,79,80]. In potatoes, the increase in H_2_O_2_ was correlated with the activation of genes involved in the active gibberellin biosynthesis, which play an important role in bud activation and elongation [69,81,82,83,84,85]. However, high doses of thiourea or H_2_O_2_ do not cause an improvement in the percentage of potato tuber sprouting [86]. It is possible that the inhibition of CAT reduces the O_2_ needed for the generation of the oxylipin family and the mobilization of storage lipids through the LOX routes.

## 3. Materials and Methods

### 3.1. Plant Material

Experiments were conducted using three commercial cultivars of *S. tuberosum* with different lengths of dormancy phase: Agata (short dormancy), Kennebec (medium dormancy) and Agria (medium-long dormancy) [36,37]. Potato tubers of the three tetraploids (2n = 4x = 48) cultivars were grown under the same conditions in an experimental field located at Xinzo de Limia (Orense, Spain). Immediately following harvest, four tubers of each cultivar were stored inside a growth chamber with a photoperiod of 16 h light/8 h dark and a temperature of 22 °C light/19 °C dark until sprouting initiation (apical bud of 2 mm), to assess the length of their dormancy period under our experimental conditions. Likewise, four tubers of each cultivar were cut separately into small pieces immediately after harvest, lyophilized and frozen at −80 °C until proteomic analysis.

### 3.2. Protein Extraction and Quantitation

Total protein was extracted from lyophilized tubers using the phenol-based extraction method as described previously by López-Pedrouso et al. [87]. Total protein concentration was assessed with the CB-X Protein Assay (Bradford) commercial kit (G-Biosciences, St. Louis, MO, USA), using a ChroMate-4300 (Awareness Technology, Palm City, FL, USA) microplate reader and Bovine Serum Albumin (BSA) protein standard for calibration curves.

### 3.3. Two-Dimensional Electrophoresis and Gel Image Analysis

Total tuber proteins (400 μg) were separated by two-dimensional electrophoresis (2-DE) according to Bernal et al. [88]. Briefly, the first-dimensional separation of proteins by isoelectric focusing (IEF) was conducted using 24 cm long immobilized pH gradient (IPG) strips (ReadyStrips™, Bio-Rad Laboratories, Hercules, CA, USA) with linear pH 4–7 on a PROTEAN^®^ IEF Cell (Bio-Rad Laboratories). The voltage was increased stepwise until reaching 70 kVh after an initial rehydration phase overnight at constant voltage of 50 V. In the second dimension, the strips equilibrated with equilibration buffers were transferred to denaturing SDS-PAGE (12%) gels (24 × 20 cm). Each gel was run on an Ettan™ DALTsix multigel electrophoresis system (GE, Healthcare, Uppsala, Sweden) at a constant electric current of 16 mA and a temperature of 25 °C until the bromophenol blue dye had just migrated off the gel bottom. Gels stained with the SYPRO Ruby protein fluorescent stain (Lonza, Rockland, ME, USA) were digitized with the Gel Doc XR + System (Bio-Rad Laboratories, Hercules, CA, USA). Gel images were analyzed with the PDQuest Advanced software v. 8.0.1 (Bio-Rad Laboratories). The automatic detection and matching of spots over samples with the PDQuest software was manually validated. Only protein spots reproducibly validated in at least three biological replicates of each cultivar were selected for further analyses. Linear IPG strips (pH 4–7) and standard molecular mass markers (15 to 200 kDa; Fermentas, Burlington, ON, Canada) loaded into a lateral well of SDS-PAGE gels were used to assess the isoelectric point (p*I*) and molecular weight (*M*_r_) of each selected spot, respectively. Spot volume quantification was carried out using volume normalization from background subtraction with the total density of those validated spots across all gels.

### 3.4. Sample Preparation for LC-MS/MS

The most intensively stained region at the center of the spots of interest was excised from the gel to maximize the protein-to-gel ratio. The excised pieces were subjected to manual in-gel tryptic digestion using the procedure developed by Shevchenko et al. [89] with minor modifications. The gel pieces were subjected to a protein reduction step with 10 mM dithiothreitol (Sigma-Aldrich, St. Louis, MO, USA) in 50 mM ammonium bicarbonate (Sigma-Aldrich) and protein alkylation with 55 mM iodoacetamide (Sigma- Aldrich) in 50 mM ammonium bicarbonate. The pieces were subsequently rinsed with 50 mM ammonium bicarbonate in 50% methanol (HPLC grade, Scharlau, Barcelona, Spain), dehydrated by adding acetonitrile (HPLC grade, Scharlau) and dried in a SpeedVac. Dried gel pieces were digested with modified porcine trypsin (Promega, Madison, WI, USA) at a final concentration of 20 ng/μL in 20 mM ammonium bicarbonate overnight at 37 °C. Peptides were extracted three times with 60% acetonitrile containing 0.5% formic acid, peptide extracts were pooled, concentrated in a SpeedVac and stored at −20 °C until mass spectrometric analysis.

### 3.5. Mass Spectrometry Analysis (LC-MS/MS)

Digested peptides were analyzed by reversed-phase liquid chromatography–tandem mass spectrometry (LC-MS/MS) using an Eksigent Technologies LC 400 system (SCIEX, Foster City, CA, USA) coupled to a high-speed TripleTOF 6600 mass spectrometer system (SCIEX) equipped with a microflow source. A combination of 5 × 0.5 mm trap column (YMC-TRIART C18, 3 mm particle size and 120 Å pore size; YMC Technologies, Teknokroma) switched on-line with the 150 × 0.30 mm reversed-phase analytical column (Chrom XP C18, 3 mm particle size and 120 Å pore size; Eksigent, SCIEX) was used. The loading pump flushed a solution of 0.1% formic acid in water at 10 µL/min. The micro-pump operated at flow rate of 5 µL/min in gradient elution mode. Mobile phases A and B were 0.1% formic acid in water and acetonitrile, respectively. A gradient ranging from 2% to 90% mobile phase B was developed over 15 min. Mobile-phase A consisted of 2% acetonitrile, 0.1% formic acid; mobile-phase B consisted of 100% acetonitrile, 0.1% formic acid. Digested peptides of each spot were resuspended in 10 µL of solution A and 4 µL were subsequently injected for protein identification. Data acquisition was performed on a TripleTOF 6600 System (SCIEX) using data-dependent workflow. Electrospray ionization (ESI) settings were: ion source gas 1 (GS1) at 25 psi, curtain gas (CUR) at 25 psi, ionspray voltage floating (ISVF) at 5500 V, and collision energy (CE) at 10 V (ion source gas 2). Analyst TF 1.7.1 software (SCIEX) was used for data acquisition. MS/MS switch criteria included 350–1400 *m/z* range, charge state of 2–5, 250 ppm mass tolerance and a count abundance threshold > 200 cps. Exclusion of former target ions was set to 15 s. Instrument calibration was automatically performed every four hours using the PepCalMix (SCIEX) reference tryptic peptides as external calibrant. Data were analyzed using the ParagonTM and Pro GroupTM algorithms for the database search and data grouping, respectively (ProteinPilotTM 5.0.1 software, SCIEX). The data search was carried out using *S. tuberosum* UniProt database. A global false discovery rate (FDR) ≤ 1% was applied using a non-linear fitting method [90].

### 3.6. Statistical Analysis

Differences in the mean volume of each protein spot between pairs of cultivars were assessed by non-parametric bootstrap confidence intervals (CIs) computed as described previously [39]. Briefly, the Monte Carlo sampling method was used to generate 2000 bootstrap samples of size n (number of replicates) = 4. The 95% bootstrap CI for the mean volume of each spot was constructed from the resampling distribution of 2000 means by the bias-corrected percentile method [39,91,92]. Adjusted 95% bootstrap CIs for multiple testing were established by the conservative Bonferroni method. Computation of CIs was carried out using R statistical software (v. 3.4.0). The relationships among the three cultivars based on differentially abundant (*p* value < 0.05) spot volumes were assessed with the principal component analysis (PCA) using XLSTAT software (v. 2014.5.03; Addinsoft, Andernach).

Changes in differentially (*p* < 0.05) represented spot volumes between pairs of cultivars were quantified with the “fold change” (*FC*) and “relative change” (*RC*) indices [39]. The *FC* value for each protein spot becomes *FC* = *y*/*x*, where *x* and *y* are the mean volumes of the spot in cultivars *x* and *y*, respectively. *FC* values of less than one were replaced by their negative reciprocals. Note that *FC* takes values of −∞ or + ∞ for unshared (or unique) spots between sample groups. Therefore, *FC* measure varies from − ∞ to + ∞. The *RC* value is calculated by *RC*= *DV*/|*DVR_max_*|, where *DV* = *y* − *x*, and *DVR_max_* is the maximum value of *DV* across significantly changing spots between all pairs of cultivars. Unlike *FC*, the *RC* measure ranges from −1.0 to + 1.0 across both shared and unshared protein spots between sample groups. Descriptive statistics were computed using IBM SPSS Statistics software (v.24; SPSS, Chicago, IL, USA).

## 4. Conclusions

Our observations suggest that comparative proteomics among potato cultivars can be an efficient experimental approach for a better understanding of the molecular mechanisms involved in dormancy length variations of great biological and commercial relevance. We have identified 11 protein/isoforms (eight non-redundant proteins) exhibiting significant changes in abundance between one or more pairs of cultivars with differential dormancy durations. This is a heterogeneous group of proteins involved in metabolism, structural, chaperone and storage functions that assist in dormancy release and tuber growth and can be candidate proteins involved in dormancy length variations. The functional analysis of the candidate proteins identified leads us to hypothesize that the oxylipin biosynthesis pathway contributes to differential dormancy duration. Further research is clearly needed using comparative proteomics over a wider range of experimental scenarios, including different tuber life cycle stages and potato varieties. The available experimental evidence indicates that 2-DE-based and gel-free MS-driven proteomic approaches provide complementary proteome profiles. Therefore, the combined use of both methodologies in pipeline studies will be an efficient resource for a more comprehensive proteome coverage linked to variable dormancy length.

## Figures and Tables

**Table 1:** Mean length of dormancy period in potato tubers of Agata, Kennebec and Agria cultivars grown under the same conditions in the present study.

Varieties	Pedigree ^1^	Origin ^1^	Mean (±SE) Dormancy Period (in Days)
Agata	BM 52/72/2206 × Sirco	Holland	15.3 (±0.3)
Kennebec	B127 × USDA X96 56	USA	53.5 (±2.0)
Agria	Quarta × Semlo	Germany	62 (±3.0)

^1^ [36,38].

**Table 3:** Fold change (*FC*) and relative change (*RC*) of differentially abundant proteins (*p* value < 0.05) between pairwise potato cultivars.

Spot Code	Protein Name	Abbrev.	Agata– Kennebec		Agata— Agria		Kennebec— Agria	
			*FC*	*RC*	*FC*	*RC*	*FC*	*RC*
1	Translationally controlled tumor protein homolog	TCTP	−1.80	−0.187	ns	ns	ns	ns
4	ADP, ATP carrier protein, mitochondrial	AAC	−∞	−0.489	−∞	−0.489	N/A	N/A
5	Heat shock 70 kDa protein, mitochondrial	mt-HSP70	ns	ns	−∞	−0.260	−∞	−0.311
6	Patatin-2-Kuras 1	PAT2-K1	ns	ns	ns	ns	+1.59	+0.116
7	ADP, ATP carrier protein, mitochondrial	AAC	+1.49	+0.090	ns	ns	ns	ns
8	Fructokinase	FRK	−2.13	−1.000	ns	ns	ns	ns
9	ADP, ATP carrier protein, mitochondrial	AAC	−1.65	−0.125	ns	ns	ns	ns
10	Patatin-05	Patatin-05	ns	ns	−∞	−0.188	−∞	−0.202
11	Linoleate 9S-lipoxygenase 2	Linolate 9S-LOX 2	−1.32	−0.097	−1.73	−0.167	−1.31	−0.070
12	Catalase isozyme 2	CAT 2	−1.49	−0.391	ns	ns	ns	ns
13	ADP, ATP carrier protein, mitochondrial	AAC	−1.36	−0.068	−1.34	−0.065	ns	ns

ns: non-significant.

## Supplementary Materials

The following supporting information can be downloaded at: https://www.mdpi.com/article/10.3390/molecules27196621/s1, Table S1: Results of protein identification for each of the 78 differentially abundant spots among potato cultivars. Table S2: Mean (±SE) volume across replicates and the corresponding 95% bootstrap CI (CL, lower bound; CU, upper bound) for spots containing a single protein with statistically significant differential abundance (*p* value < 0.05) between pairs of potato cultivars (Agata, AGA; Kennebec, KEN; Agria, AGR) at the dormancy stage. Figure S1: 2-DE profiles across four biological replicates (1–4) of Agata, Kennebec and Agria potato cultivars at the endodormancy stage.

## Sample Availability

Not available.

## Abbreviations

2-DE	Two-dimensional electrophoresis
9-HPODE	(9S,10E,12Z)-9-hydroperoxy-10,12-octadecadienoic acid
AAC	ADP, ATP carrier
ABA	Abscisic acid
ATP	Adenosine triphosphate
CAT	Catalase
CE	Collision energy
CI	Confidence interval
CIPC	Chlorpropham
CUR	Curtain gas
ESI	Electrospray ionization
*FC*	Fold change
FDR	False discovery rate
FRK	Frucktokinase
H_2_O_2_	Hydrogen peroxide
IEF	Isoelectric focusing
IPG	Immobilized pH gradient
ISVF	Ionspray voltage floating
LC—MS/MS	Liquid chromatography-tandem mass spectrometry
LOX	Lypoxygenase
*M*_r_	Relative molecular mass
MS	Mass spectrometry
O_2_-	Superoxide
p*I*	Isoelectric point
PUFAS	Polyunsaturated fatty acids
*RC*	Relative change
ROS	Reactive oxygen species
SDS-PAGE	Sodium dodecyl sulphate–polyacrylamide gel electrophoresis
StAOS3	Allene oxide synthase 3
TCTP	Translationally controlled tumor protein homolog
