# Supplementary material for: Comparative Proteomics of Potato Cultivars with a Variable Dormancy Period
Source: Molecules. 2022 Oct 5;27(19):6621. doi: 10.3390/molecules27196621 (PMC9573702; doi:10.3390/molecules27196621)
Supplement: Supplementary file 1 [file molecules-27-06621-s001.zip › Suppl. Figure S1.pdf]

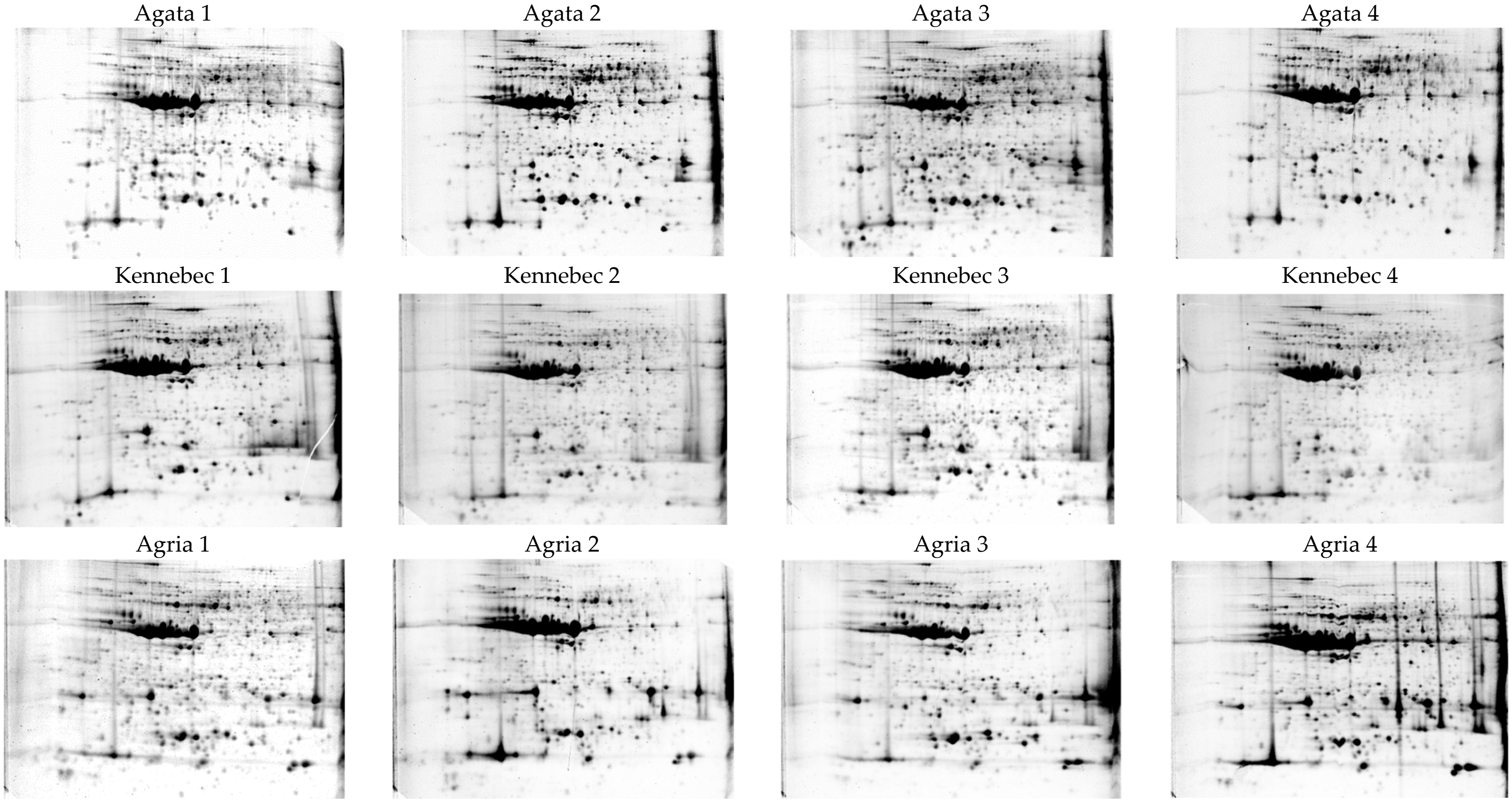

Figure S1. 2-DE profiles across four biological replicates (1-4) of Agata, Kennebec and Agria potato cultivars at the endodormancy stage.
