# Supplementary material for: Comparative Proteomics of Potato Cultivars with a Variable Dormancy Period
Source: Molecules. 2022 Oct 5;27(19):6621. doi: 10.3390/molecules27196621 (PMC9573702; doi:10.3390/molecules27196621)
Supplement: Supplementary file 1 [file molecules-27-06621-s001.zip › Suppl. Table S2.pdf]

Table S2. Mean (± SE) volume across replicates and the corresponding 95% bootstrap CI (CL, lower bound; CU, upper bound) for spots containing a single protein with statistically significant differential abundance (*P*-value < 0.05) between pairs of potato cultivars (Agata, AGA; Kennebec, KEN; Agria, AGR) at the dormancy stage.

Spot code	Agata		Kennebec		Agria		*P*-value		
	Volume	95% Bootstrap CI (CL,CU)	Volume	95% bootstrap CI (CL,CU)	Volume	95% bootstrap CI (CL,CU)	AGA-KEN	AGA-AGR	KEN-AGR
1	1217 ± 137	(1057, 1627)	676 ± 53	(568, 773)	1445 ± 363	(678, 1997)	< 0.05		
4	1414 ± 563	(625, 3054)					< 0.05	< 0.05	
5	752 ± 75	(616, 945)	899 ± 252	(445, 1544)				< 0.05	< 0.05
6	665 ± 114	(533, 863)	570 ± 57	(451, 659)	906 ± 110	(705, 1190)			< 0.05
7	533 ± 74	(386, 623)	792 ± 54	(693, 924)	726 ± 148	(563, 1014)	< 0.05		
8	5450 ± 457	(4716, 6731)	2559 ± 261	(2107, 3194)	4514 ± 858	(2695, 5706)	< 0.05		
9	915 ± 60	(800, 1057)	553 ± 111	(325, 738)	845 ± 152	(619, 1282)	< 0.05		
10	543 ± 65	(414, 654)	583 ± 31	(535, 671)				< 0.05	< 0.05
11	1149 ± 45	(1077, 1270)	868 ± 62	(764, 1026)	665 ± 36	(591, 724)	< 0.05	< 0.05	< 0.05
12	3448 ± 190	(3078, 3817)	2317 ± 178	(1945, 2628)	2607 ± 421	(1811, 3510)	< 0.05		
13	744 ± 34	(689, 839)	546 ± 53	(437, 632)	557 ± 55	(441, 636)	< 0.05	< 0.05	
